# Diagnostic performance of bpMRI versus mpMRI and AI-assisted bpMRI in prostate cancer detection: a multi-reader study

**DOI:** 10.1007/s11547-026-02252-7

**Published:** 2026-06-29

**Authors:** Luca Russo, Silvia Bottazzi, Luca D’Erme, Giacomo Avesani, Valerio Di Paola, Salvatore Persiani, Alessandra Iacono, Ina Isufi, Lucy Davies, Zobair Arya, Matteo Mancino, Francesca Lia, Giuseppe Pallotta, Maria Chiara Sighinolfi, Francesco Pierconti, Bernardo Rocco, Evis Sala, Benedetta Gui

**Affiliations:** 1 Fondazione Policlinico Universitario A. Gemelli IRCCS, Largo Agostino Gemelli 8, 00168 Rome, Italy; 2 Università Cattolica del Sacro Cuore, Rome, Italy; 3 Lucida Medical Ltd, Cambridge, UK

**Keywords:** Prostatic neoplasms, Magnetic resonance imaging, Artificial intelligence, Decision support

## Abstract

**Purpose:**

To compare biparametric MRI (bpMRI) and multiparametric MRI (mpMRI) for detecting clinically significant prostate cancer (csPCa), and to assess the impact of artificial intelligence (AI)–assisted bpMRI on diagnostic performance and biopsy-related outcomes in readers with different expertise.

**Material and methods:**

In this retrospective multi-reader study, 173 men referred for prostate mpMRI were evaluated by five radiologists (two experts, three basic readers) who scored bpMRI and mpMRI using PI-RADS v2.1. After a 45-day wash-out, the same readers reinterpreted bpMRI with concurrent AI decision support (available for 127 cases). Diagnostic performance for csPCa (ISUP ≥ 2) and benefit-to-harm ratios were compared across protocols and reader groups.

**Results:**

Mean area under the receiver operating characteristic curve was 0.820 for bpMRI and 0.819 for mpMRI (difference 0.001), indicating comparable diagnostic performance. In the AI subset, AI increased mean specificity from 59.7% to 67.8% while reducing sensitivity from 90.2% to 84.5%. Biopsy selectivity and efficiency improved (3.9 to 4.6 and 1.7–1.9), particularly in basic readers. The effect was significant in basic readers (McNemar *p* = 0.012 for sensitivity, *p* < 0.001 for specificity) but not in experts.

**Conclusion:**

bpMRI showed comparable performance to mpMRI for csPCa detection in routine practice. AI-assisted bpMRI improved biopsy efficiency in less-experienced readers at the cost of reduced sensitivity, warranting careful consideration of the trade-off between missed cancers and avoided unproductive biopsies.

## Introduction

Prostate MRI is the key imaging test for men with suspected clinically significant prostate cancer (csPCa), improving both biopsy targeting and avoidance of unnecessary procedures [1–4]. The standard protocol is multiparametric MRI (mpMRI), which combines T2-weighted imaging (WI), diffusion-weighted imaging (DWI), and dynamic contrast-enhanced (DCE) imaging [5, 6]. However, DCE increases examination time and costs, while its incremental contribution within PI-RADS is limited, mainly acting in selected scenarios [7, 8]. Recent studies, including level 1B evidence, have suggested that high-quality biparametric MRI (bpMRI), relying on T2-WI and DWI alone, may achieve diagnostic performance comparable to mpMRI for csPCa detection, but most data derive from single-reader or tightly controlled settings and do not address how bpMRI performs across readers with heterogeneous expertise in routine practice [9–11].

In parallel, AI tools for prostate MRI have shown performance approaching that of subspecialist radiologists and may help reporting and support biopsy decision-making [12, 13]. To date, validation has largely focused on mpMRI and on conventional diagnostic metrics such as sensitivity, specificity and AUC [14, 15]. Much less is known about the behaviour of AI when integrated into a bpMRI-based workflow, its impact on readers with different levels of experience, and how any changes in diagnostic performance translate into the balance between cancer detection and biopsy decision [16]. Recently proposed benefit-to-harm ratios offer a way to quantify this balance more directly [17].

In this context, we conducted a retrospective, two-phase multi-reader study in a consecutive referral cohort. Our primary objective was to compare the diagnostic performance of bpMRI and mpMRI for csPCa detection at the patient level, using AUC as the primary endpoint. Once comparable performance of bpMRI and mpMRI had been established, we sought to evaluate the effect of an AI-assisted bpMRI workflow on diagnostic accuracy and on benefit-to-harm ratios, overall and stratified by reader expertise.

## Methods

### Study population

The study was approved by the institutional review board (ID: 7680). Consecutive men referred for prostate mpMRI between January 2020 and December 2024 after identification of elevated PSA (PSA > 3 ng/mL), with or without abnormal digital rectal examination (DRE), were identified. Examinations were included when the mpMRI study was completed at our institution and when a reference standard was available either as histopathology (systematic and/or targeted biopsy, or radical prostatectomy) or as clinical/biochemical/radiological follow-up of at least 12 months. Exclusion criteria were: non-diagnostic scans according to PI-QUAL v2 (score 1) [18], incomplete mpMRI protocol, patients who received prior biopsy or treatment for prostate cancer (androgen deprivation, focal therapy, prostatectomy, or radiotherapy).

The reference standard was defined hierarchically. In clinical practice, biopsy was recommended for men with PI-RADS ≥ 3 lesions, or for men with PI-RADS 2 and high clinical suspicion (e.g., PSA density > 0.15 ng/mL^2^ or rising PSA trend), at the discretion of the referring urologist. When systematic and/or MRI-targeted biopsy was available after MRI, the highest ISUP grade group at biopsy was used. When radical prostatectomy was performed, the highest ISUP grade group at prostatectomy replaced biopsy findings. In the absence of tissue diagnosis, absence of clinically significant prostate cancer was inferred from at least 12 months of clinical, biochemical, and/or MRI follow-up showing stable or decreasing PSA values and no imaging progression. Clinically significant prostate cancer was defined as ISUP grade group ≥ 2 [19].

### MRI acquisition

All MRI examinations were performed on 1.5 T or 3 T scanners from the same vendor (GE Healthcare, Chicago, IL, USA) following institutional protocols compliant with PI-RADS v2.1 (detailed sequence parameters are provided in Online Resource, 1) [5]. The routine mpMRI protocol comprised high-resolution T2-WI (axial, sagittal and coronal planes), DWI with ADC maps, and DCE imaging. All exams were anonymised and uploaded to Collective Minds Research platform (Collective Minds, Danderyd, Sweden).

### Study design and image interpretation

This was an observational, retrospective, two-phase multi-reader study conducted at a single Institution. Image interpretation was carried out on Collective Minds Research platform in two sessions separated by a 45-day wash-out. Five radiologists participated: two expert readers (each having reported > 1000 prostate MRI cases) and three basic readers (200–1000 cases in supervised settings), classified according to ESUR/ESUI criteria [20]. In the first session each reader independently reviewed the bpMRI dataset and assigned a PI-RADS v2.1 score; immediately after this assessment the DCE images of the same patient were unblinded and the reader could confirm or modify the judgement, producing the mpMRI PI-RADS score. In the second session, conducted after a wash-out period of 45 days on the same cases, the same readers reassessed the examinations with the output of a commercial AI system (Pi™ v. 2.4.4, Lucida Medical, Cambridge, UK) available together with bpMRI. AI reports were available for 127 of 173 examinations; the remaining 46 could not be processed owing to a technical incompatibility between the AI software and the scanner identification metadata in the DICOM headers, unrelated to clinical or image-quality factors. The AI system provided automated lesion detection, segmentation, and a continuous index score (0–5) (Online Resource, 2). Readers produced the AI-assisted bpMRI score in a concurrent decision-support setting, with the AI output displayed alongside the bpMRI images; readers were free to agree with, modify, or disregard the AI suggestion [12]. Readers were blinded to histopathology and follow-up. For each session and reader, the highest PI-RADS was used as patient-level score.

### Study outcomes and statistical analysis

The primary outcome was comparing diagnostic performance of bpMRI and mpMRI for the detection of csPCa, defined as ISUP grade group ≥ 2, at the main diagnostic threshold PI-RADS ≥ 3. The primary endpoint was the area under the receiver-operating-characteristic curve (AUC) derived from ordinal PI-RADS scores (1–5), evaluated at the main diagnostic threshold of PI-RADS ≥ 3. mpMRI was taken as the reference imaging strategy, and non-inferiority of bpMRI was tested against a 10% absolute margin on AUC (bpMRI – mpMRI ≥ –0.10). A 10% absolute margin was selected a priori as a clinically pragmatic threshold, in keeping with margins adopted in previous bpMRI-versus-mpMRI comparative studies [15], and was considered to represent the largest reduction in AUC that would still be acceptable in exchange for the shorter acquisition time and the avoidance of gadolinium afforded by bpMRI. For each reader and protocol, ROC curves were constructed and AUCs estimated; the difference in AUC between bpMRI and mpMRI and its 95% confidence interval were obtained using multi-reader–multicase methods. bpMRI was considered non-inferior if the lower confidence bound for the AUC difference was greater than –0.10 (corresponding to a one-sided α of 0.025).

Secondary outcomes were to compare diagnostic performance between bpMRI and AI-assisted bpMRI, and to assess whether AI produced different effects in expert and non-expert readers. For each reader and for each strategy (bpMRI and AI-assisted bpMRI) we calculated, at PI-RADS ≥ 3, PI-RADS ≥ 4, and at a composite rule (PI-RADS 3 with PSA density > 0.15 ng/mL^2^ or PI-RADS ≥ 4), the following diagnostic metrics: sensitivity, specificity, positive predictive value (PPV), negative predictive value (NPV) and accuracy. Paired proportions were compared with McNemar’s test. Expert and non-expert readers were analysed separately, and an interaction term between imaging strategy and reader expertise was used to test for differential AI effect.

Also, benefit-to-harm ratios were defined according to Schoots et al. [17]. For men undergoing biopsy (i.e. men who would have been referred to biopsy by that MRI strategy), we calculated: biopsy selectivity (GG ≥ 2/GG1 ratio) and biopsy efficiency (GG ≥ 2/[GG1 + benign]). For men avoiding biopsy (i.e. men who would not have been referred to biopsy by that MRI strategy), we calculated: safe avoidance (avoided biopsy/undetected GG ≥ 2) and selective avoidance (avoided biopsy/benign biopsy). Additionally, the absolute number of missed csPCa (false negatives at PI-RADS ≥ 3) was recorded for each reader and strategy to quantify the clinical cost of specificity-oriented shifts in the diagnostic operating point. All ratios were computed separately for each MRI strategy, for each threshold, and then repeated in the two reader-expertise subgroups. Inter-reader agreement was assessed using Fleiss’ κ at the binary threshold of PI-RADS ≥ 3 versus < 3, with 95% confidence intervals obtained by bootstrap resampling (2,000 iterations). Exploratory analyses were repeated for PI-RADS ≥ 4 and for the composite PSAd-based rule. All tests were two-sided except for the non-inferiority test, which was one-sided at α = 0.025. No formal a priori sample size calculation was performed for the non-inferiority comparison; the analysis was conducted on all consecutive eligible patients in the study period, and the non-inferiority result should therefore be interpreted as exploratory rather than confirmatory. Analyses were performed in R (R Foundation for Statistical Computing, Vienna, Austria), and p values < 0.05 were considered statistically significant.

## Results

### Patient population

**Fig. 1:**
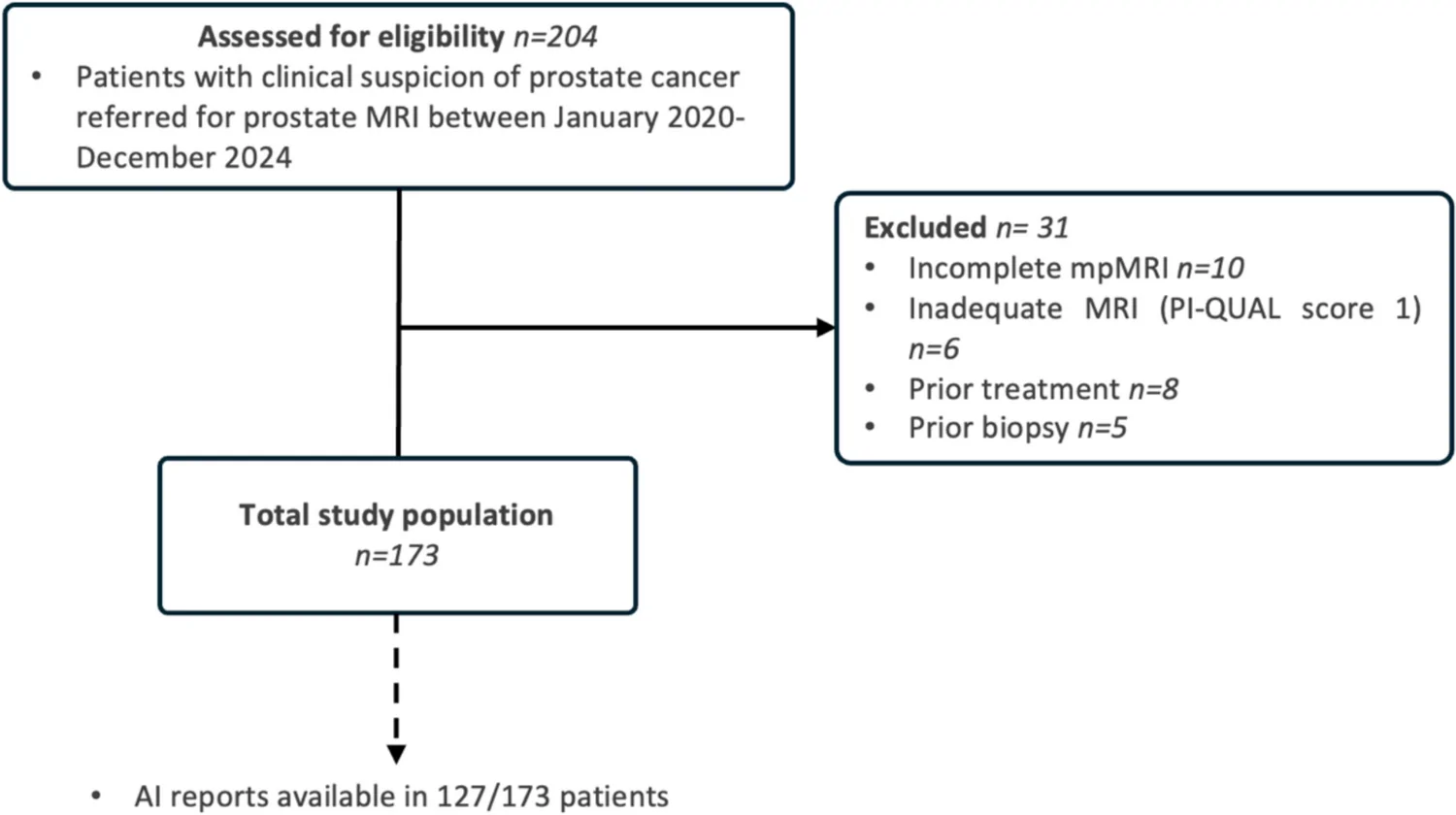
Flow diagram of the study population

Overall, 173 men met the inclusion criteria and were included in the analysis (Fig. 1). Median age was 67 years (IQR, 58–71), with a median prostate volume of 57.7 mL (IQR, 42.7–77.4), median PSA of 7.3 ng/mL (IQR, 5.3–10.7) and median PSA density of 0.12 ng/mL^2^ (IQR, 0.09–0.21). The distribution of worst ISUP grade among histology-proven cases was benign in 66 (42.0%), ISUP 1 in 22 (14.0%), ISUP 2 in 28 (17.8%), ISUP 3 in 19 (12.1%), ISUP 4 in 12 (7.6%) and ISUP 5 in 10 (6.4%). Detailed baseline characteristics of the study population were reported in Table 1.

**Table 1:** Characteristics of the study population

	All cases	AI report available	AI report not available
	*n* = 173	*n* = 127	*n* = 46
Age, years [median (IQR)]	67 (58–71)	66 (59–71)	68 (58–71)
Prostate volume, ml [median (IQR)]	57.7 (42.7–77.4)	56.4 (43.4–77.3)	59.1 (42.7–77.2)
PSA, ng/mL [median (IQR)]	7.3 (5.3–10.7)	7.5 (5.1–11.0)	7.4 (5.3–10.8)
PSAd, ng/mL^2^ [median (IQR)]	0.12 (0.09–0.21)	0.13 (0.09–0.21)	0.12 (0.09–0.20)
csPCa cases, no. (%)	69 (39.9)	53 (41.7)	16 (34.8)
Benign or insignificant PCa cases (%)	106 (61.3)	74 (58.3)	32 (64.4)
No histology, no (%) ^a^	16 (9.2)	14 (11.0)	2 (4.3)
MRI-TRUS fusion biopsy, no (%) ^b^	116 (67.1)	81 (63.8)	35 (76.1)
Radical prostatectomy, no (%)	41 (23.7)	32 (25.2)	9 (19.6)
Worst ISUP score ^c^			
Benign	66 (42.0)	51 (40.2)	15 (32.6)
GG1	22 (14.0)	18 (14.2)	4 (8.7)
GG2	28 (17.8)	21 (16.5)	7 (15.2)
GG3	19 (12.1)	13 (10.2)	6 (13.0)
GG4	12 (7.6)	10 (7.9)	2 (4.3)
GG5	10 (6.4)	9 (7.1)	1 (2.2)

^a^12-months follow-up

^b^Target + systematic biopsy

^c^Defined on a per-patient level in patients with histological results (biopsy or prostatectomy)

### Comparison between bpMRI and mpMRI

**Fig. 2:**
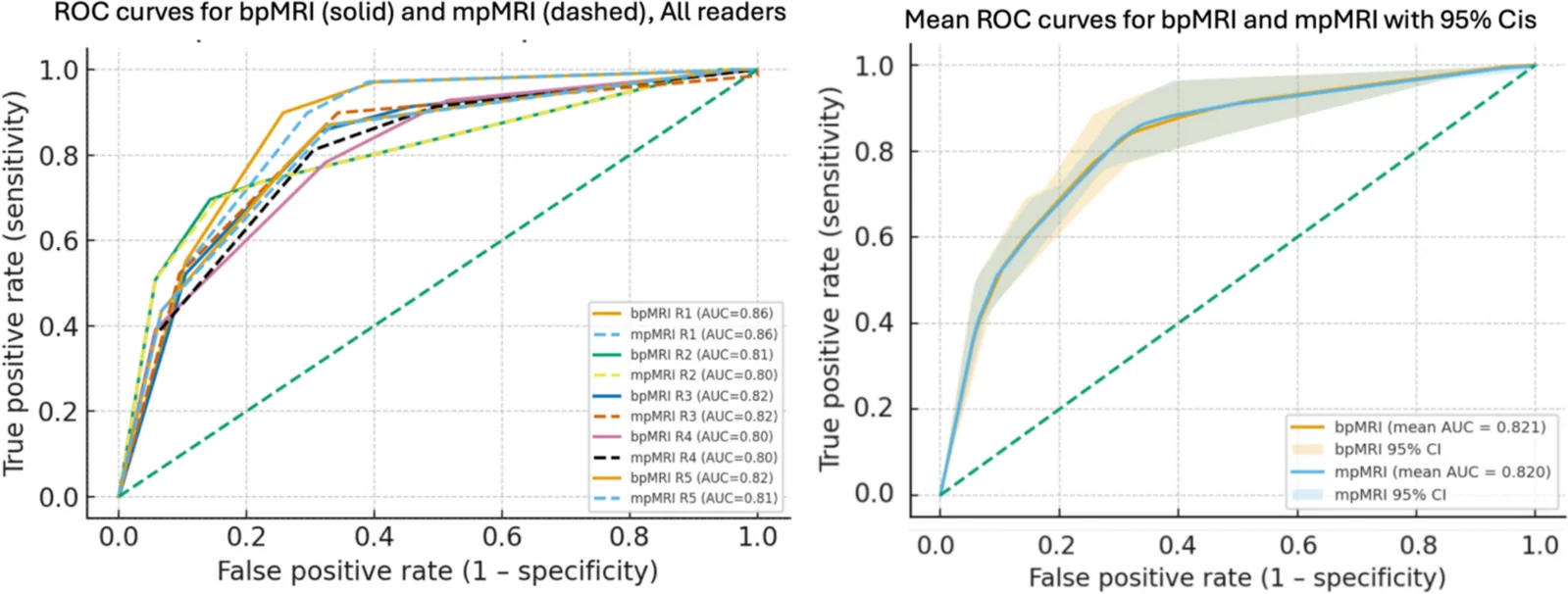
ROC curves for bpMRI (solid lines) and mpMRI (dashed lines) for each reader (left) and mean ROC curves with 95% confidence intervals (right) for detection of clinically significant prostate cancer (csPCa) (*n* = 173)

All 173 examinations were available for the primary comparison between bpMRI and mpMRI. At the main diagnostic threshold of PI-RADS ≥ 3, the average AUC across readers was 0.820 for bpMRI and 0.819 for mpMRI, with an absolute difference of 0.1% (bpMRI–mpMRI, 0.1% [95% CI –0.6% to 0.7%]) (Fig. 2). Mean sensitivity for csPCa was 88.4% with bpMRI and 88.1% with mpMRI (difference 0.3% [95% CI –0.5% to 1.1%]), while mean specificity was 61.5% and 61.9%, respectively (difference –0.4% [95% CI –1.7% to 1.0%]; Table 2). Mean PPV was 60.9% with both bpMRI and mpMRI, while mean NPV was 89.8% and 89.5%. A non-inferiority analysis indicated that bpMRI performed comparably to mpMRI for csPCa detection (non-inferiority p < 0.001); however, in the absence of a formal sample size calculation, this result should be considered supportive rather than confirmatory. The number of csPCa diagnoses was very similar with the two strategies, with a median of 63/69 cancers detected per reader with both bpMRI (IQR, 60–64) and mpMRI (IQR, 60–63), and a comparable yield of ISUP 1 cancers (median 17 [IQR, 16–17] vs 16 [IQR, 16–16]). Patient-level PI-RADS categories were the same on bpMRI and mpMRI in 829/865 readings (95.8%). Inter-reader agreement at PI-RADS ≥ 3 was moderate and similar for both protocols: Fleiss’ κ = 0.53 (95% CI, 0.45–0.61) for bpMRI and 0.54 (95% CI, 0.46–0.61) for mpMRI.

**Table 2:** Per-reader diagnostic performance of bpMRI and mpMRI for detection of csPCa at PI-RADS ≥ 3 (patient level, *n* = 173)

Reader	bpMRI AUC	mpMRI AUC	bpMRI sensitivity (%)	mpMRI sensitivity (%)	bpMRI specificity (%)	mpMRI specificity (%)	bpMRI PPV (%)	mpMRI PPV (%)	bpMRI NPV (%)	mpMRI NPV (%)	bpMRI accuracy (%)	mpMRI accuracy (%)
R1	0.864	0.857	97.1	97.1	60.0	61.0	61.5	62.0	96.9	97.0	74.7	75.3
R2	0.806	0.805	73.9	73.9	77.1	77.1	68.0	68.0	81.8	81.8	75.9	75.9
R3	0.817	0.820	91.3	91.3	54.3	54.3	56.8	56.8	90.5	90.5	69.0	69.0
R4	0.799	0.805	92.8	91.3	48.6	50.5	54.2	54.8	91.1	89.8	66.1	66.7
R5	0.818	0.814	87.0	87.0	67.6	66.7	63.8	63.2	88.8	88.6	75.3	74.7
Mean ± SD	0.820 ± 0.026	0.819 ± 0.021	88.4 ± 8.9	88.1 ± 8.7	61.5 ± 11.2	61.9 ± 10.5	60.9 ± 5.5	60.9 ± 5.3	89.8 ± 5.4	89.5 ± 5.4	72.2 ± 4.4	72.3 ± 4.2

AUC, area under the ROC curve; PPV, positive predictive value; NPV, negative predictive value. Sensitivity, specificity, accuracy, PPV and NPV calculated at PI-RADS ≥ 3 vs csPCa (ISUP ≥ 2)

### Diagnostic performance of bpMRI versus AI-assisted bpMRI

In the subset with AI reports available (127/173 men; 53/127 [41.7%] with csPCa), we compared diagnostic performance of bpMRI and AI-assisted bpMRI (Table 3). At PI-RADS ≥ 3, across all readers AI assistance shifted the balance towards higher specificity at the cost of sensitivity. Mean sensitivity decreased from 90.2% (± 6.6) to 84.5% (± 9.3), while mean specificity increased from 59.7% (± 10.6) to 67.8% (± 6.4). Overall accuracy rose slightly from 72.4% (± 5.0) to 74.8% (± 2.5). In expert readers, sensitivity and specificity remained high and similar with and without AI: sensitivity 89.6% (± 12.0) (bpMRI) vs 86.8% (± 13.3) (AI-assisted bpMRI); specificity 68.2% (± 8.6) (bpMRI) vs 68.9% (± 5.7) (AI-assisted bpMRI). In basic readers, mean specificity increased with AI assistance from 54.1% (± 8.2) to 67.1% (± 7.9) and accuracy from 69.3% (± 3.6) to 73.8% (± 2.4), while sensitivity fell from 90.6% (± 3.8) to 83.0% (± 8.6). AI-assisted sensitivity was significantly lower than bpMRI sensitivity in basic readers (pooled McNemar p = 0.012) but not in experts (*p* = 0.453); conversely, AI-assisted specificity was significantly higher in basic readers (*p* < 0.001) but not in experts (*p* = 1.000). The mean number of missed csPCa per reader increased from 5.2 ± 3.5 with bpMRI to 8.2 ± 4.9 with AI-assisted bpMRI; in basic readers, the increase was from 5.0 ± 2.0 to 9.0 ± 4.6. Similar patterns were seen at PI-RADS ≥ 4 and for the composite PSAd-based threshold, with AI consistently improving specificity and accuracy in basic readers and producing minimal change in expert readers (Online Resource, 3). Inter-reader agreement with AI-assisted bpMRI was moderate (Fleiss’ κ = 0.61; 95% CI, 0.52–0.69), comparable to bpMRI alone in the same subset (0.58; 95% CI, 0.48–0.67).

**Table 3:** Diagnostic performance of bpMRI versus AI-assisted bpMRI at PI-RADS ≥ 3 (AI subset, n = 127)

Reader / group	bpMRI sensitivity (%)	AI-assisted sensitivity (%)	p (sens)	bpMRI specificity (%)	AI-assisted specificity (%)	p (spec)	bpMRI PPV (%)	AI-assisted PPV (%)	bpMRI NPV (%)	AI-assisted NPV (%)	bpMRI accuracy (%)	AI-assisted accuracy (%)
R1 (expert)	98.1	96.2	1.000	62.2	64.9	0.791	65.0	66.2	97.9	96.0	77.2	78.0
R2 (expert)	81.1	77.4	0.688	74.3	73.0	1.000	69.4	67.2	84.6	81.8	77.2	74.8
R3 (basic)	94.3	84.9	0.062	50.0	70.3	< *0.001*	57.5	67.2	92.5	86.7	68.5	76.4
R4 (basic)	90.6	90.6	1.000	48.6	58.1	0.167	55.8	60.8	87.8	89.6	66.1	71.7
R5 (basic)	86.8	73.6	*0.039*	63.5	73.0	0.092	63.0	66.1	87.0	79.4	73.2	73.2
All readers (mean ± SD)	90.2 ± 6.6	84.5 ± 9.3	*0.006*	59.7 ± 10.6	67.8 ± 6.4	< *0.001*	62.1 ± 5.5	65.5 ± 2.7	90.0 ± 5.3	86.7 ± 6.6	72.4 ± 5.0	74.8 ± 2.5
Experts (mean ± SD)	89.6 ± 12.0	86.8 ± 13.3	0.453	68.2 ± 8.6	68.9 ± 5.7	1.000	67.2 ± 3.1	66.7 ± 0.7	91.2 ± 9.4	88.9 ± 10.0	77.2 ± 0.0	76.4 ± 2.2
Basic readers (mean ± SD)	90.6 ± 3.8	83.0 ± 8.6	*0.012*	54.1 ± 8.2	67.1 ± 7.9	< *0.001*	58.8 ± 3.8	64.7 ± 3.4	89.1 ± 3.0	85.2 ± 5.2	69.3 ± 3.6	73.8 ± 2.4

Experts = R1–R2; basic readers = R3–R5. Metrics at PI-RADS ≥ 3 vs csPCa (ISUP ≥ 2). PPV, positive predictive value; NPV, negative predictive value. p (sens) and p (spec) = McNemar p-values comparing bpMRI vs AI-assisted bpMRI; group-level p-values obtained by pooling discordant pairs across readers

### Benefit-to-harm ratio analysis

For all readers combined, biopsy selectivity for csPCa over GG 1 improved from 3.9 ± 0.9 with bpMRI to 4.6 ± 1.2 with AI-assisted bpMRI, and biopsy efficiency increased from 1.7 ± 0.4 to 1.9 ± 0.2 (Table 4). Selective avoidance of unproductive biopsies rose from 3.3 ± 1.7 to 4.6 ± 1.7. In expert readers, biopsy selectivity increased from 4.7 ± 1.0 to 5.4 ± 2.1, biopsy efficiency remained similar (2.1 ± 0.3 vs 2.0 ± 0.1), and selective avoidance changed little (4.5 ± 2.0 vs 4.3 ± 0.6), while safe avoidance decreased but remained good (15.2 ± 13.8), reflecting only one or two extra missed csPCa. In basic readers, biopsy selectivity increased from 3.4 ± 0.2 to 4.1 ± 0.2, biopsy efficiency from 1.4 ± 0.2 to 1.8 ± 0.3, and selective avoidance from 2.5 ± 1.2 to 4.9 ± 2.3, while safe avoidance decreased from 9.7 ± 3.1 to 7.3 ± 2.4. At PI-RADS ≥ 4 and at the composite PSAd-based rule, AI again increased selective avoidance, particularly in basic readers (Online Resource, 4).

**Table 4:** Benefit-to-harm ratios at PI-RADS ≥ 3 for bpMRI versus AI-assisted bpMRI

Metric	All readers bpMRI	All readers AI-assisted bpMRI	Expert readers bpMRI	Expert readers AI-assisted bpMRI	Basic readers bpMRI	Basic readers AI-assisted bpMRI
Biopsy selectivity	3.9 ± 0.9	4.6 ± 1.2	4.7 ± 1.0	5.4 ± 2.1	3.4 ± 0.2	4.1 ± 0.2
Biopsy efficiency	1.7 ± 0.4	1.9 ± 0.2	2.1 ± 0.3	2.0 ± 0.1	1.4 ± 0.2	1.8 ± 0.3
Safe avoidance	16.6 ± 17.2	10.5 ± 8.3	26.8 ± 28.6	15.2 ± 13.8	9.7 ± 3.1	7.3 ± 2.4
Selective avoidance	3.3 ± 1.7	4.6 ± 1.7	4.5 ± 2.0	4.3 ± 0.6	2.5 ± 1.2	4.9 ± 2.3
Missed csPCa	5.2 ± 3.5	8.2 ± 4.9	5.5 ± 6.4	7.0 ± 7.1	5.0 ± 2.0	9.0 ± 4.6

Selectivity = csPCa / GG1 among biopsied; efficiency = csPCa / (GG1 + benign); safe avoidance = avoided biopsies / undetected csPCa; selective avoidance = avoided biopsies / benign or GG1 biopsies; missed csPCa = false-negative cases (csPCa not referred to biopsy by that strategy)

## Discussion

In this routine-care multi-reader cohort, bpMRI showed comparable patient-level discrimination for csPCa to that of mpMRI. Moreover, adding AI to bpMRI can refine biopsy decisions, particularly for less-experienced readers. At the primary threshold of PI-RADS ≥ 3, bpMRI and mpMRI were essentially indistinguishable in terms of AUC, sensitivity and specificity and produced almost identical csPCa yields per reader, suggesting that in our setting contrast can be omitted without measurable loss of diagnostic accuracy. The AI analysis adds a second, clinically relevant layer. In the AI-available subset, AI systematically moved the diagnostic operating point towards a more conservative use of biopsy: sensitivity decreased, but specificity and overall accuracy increased, and the biopsy pathways became more selective and efficient. For expert readers, these shifts were small; they already operated near an “optimal” point on the ROC curve. For less-experienced readers, AI had a more marked effect: specificity rose by approximately 13 percentage points, accuracy improved, and biopsy selectivity and efficiency improved substantially. From a clinical decision-making perspective, AI support reduced the frequency of recommended biopsy by less-experienced radiologists, while increasing the proportion of biopsies yielding clinically significant cancer.

Our bpMRI–mpMRI findings are consistent with current evidence. Several single-centre and multicentre studies, including large, randomised data, have reported comparable performance for bpMRI and mpMRI, suggesting that DCE has limited incremental value when T2-WI and DWI are optimised [10, 21–23]. Our results extend this by supporting, across readers with different experience levels, that omitting contrast in our institution does not impair csPCa detection. The added value of our approach lies in combining multi-reader design with patient-level analysis, reflecting real-world reporting more closely than single-reader or lesion-only studies. Recent work by Gelikman et al. evaluated a deep-learning model across six radiologists, showing that AI assistance improved lesion-level PPV and substantially increased Inter-reader agreement for PI-RADS scoring, while patient-level AUC for csPCa remained unchanged and lesion-level sensitivity decreased slightly [16]. Our results are complementary: in a different cohort and with a different AI system, we likewise observed improved accuracy and reduced sensitivity with AI support, but we additionally quantified how these shifts translate into biopsy decision-making. Ponsiglione et al. recently reported concordant findings using a different CE-marked AI system as concurrent decision support for mpMRI across six readers: experts were unaffected by AI, while less-experienced readers showed reduced sensitivity without a meaningful gain in specificity [24]. In our study, AI similarly had no significant effect on expert performance, but basic readers traded sensitivity for a substantial increase in specificity (+ 13 pp), resulting in improved biopsy selectivity and efficiency. Of note, in the PAIR-1 multicentre validation by Giganti et al., the same AI system (Pi v2.4) operating as a fully automated standalone tool achieved 95% sensitivity and 67% specificity for csPCa detection [15]. The lower sensitivity and higher specificity observed in the present concurrent-use setting likely reflect the anchoring effect of AI on human decision-making, which shifts the combined operating point towards a more conservative threshold, and suggests that the mode of AI integration (standalone vs concurrent decision support) may substantially influence the resulting diagnostic profile.

**Fig. 3:**
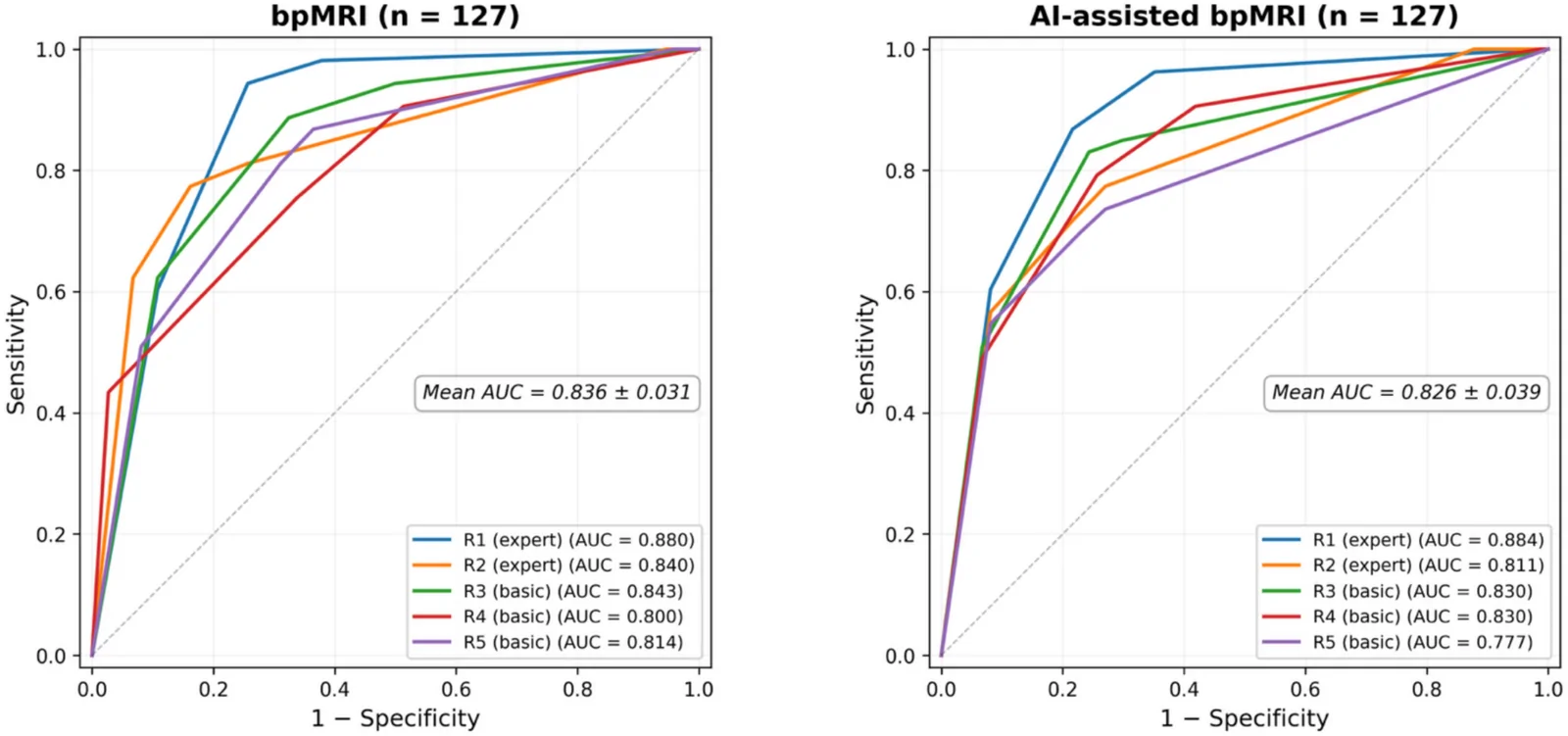
ROC curves for bpMRI (left) and AI-assisted bpMRI (right) for each reader in the AI subset (*n* = 127). Expert readers (R1, R2) are shown in blue and orange; basic readers (R3–R5) in green, red, and purple

The benefit-to-harm framework was adopted from the Schoots et al. meta-analysis, which emphasised that traditional metrics (e.g. sensitivity and specificity) do not fully capture the clinical impact of MRI-based pathways [17]. Composite ratios such as biopsy selectivity and efficiency, and safe versus selective avoidance, more directly quantify outcomes relevant to patients, namely the balance between significant cancers detected, unnecessary biopsies performed, and procedures safely avoided. In our cohort, AI-assisted bpMRI increased biopsy selectivity and efficiency and enhanced selective avoidance, particularly in basic readers, while safe-avoidance values decreased but remained within a clinically interpretable range. Taken together, these findings indicate that AI could optimise an existing MRI-based pathway by better discriminating which men should undergo biopsy and which can safely defer it. However, the accompanying decrease in NPV — from 90.0% to 86.7% overall and from 89.1% to 85.2% in basic readers — should be weighed against these gains, as it implies a slightly larger proportion of undetected csPCa among men not referred for biopsy (Fig. 3).

**Fig. 4:**
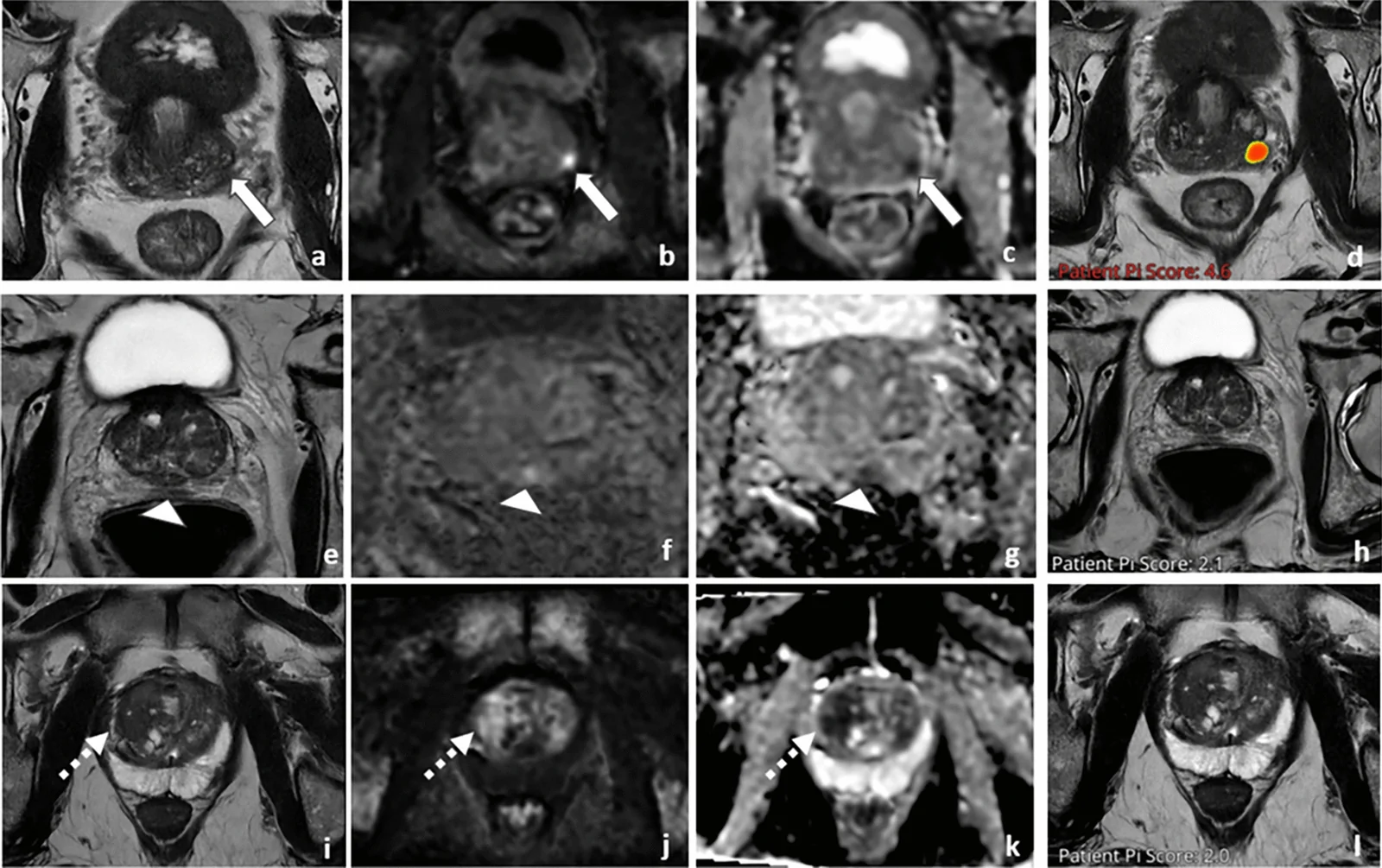
Three representative cases illustrating concordant and discordant AI-reader interaction. Case 1: Axial T2-WI (**a**), high b-value DWI (**b**), and ADC map (**c**) show a lesion in the left posterolateral peripheral zone at the base (arrows), scored PI-RADS 4 by all readers on bpMRI; the AI system confirmed a high-risk finding with a Pi score of 4.6 (d, colour overlay on T2-weighted image). Targeted biopsy yielded ISUP GG3 (Gleason 4 + 3). Case 2: Axial T2-WI (**e**), high b-value DWI (**f**), and ADC map (**g**) demonstrate a subtle finding in the right posteromedial peripheral zone at the base (arrowheads), scored PI-RADS 3 by a basic reader on bpMRI. On AI-assisted reading, the AI system did not detect a focal lesion (Pi score 2.1; h), and the reader downgraded the score to PI-RADS 2. Targeted biopsy revealed ISUP GG2 (Gleason 3 + 4), illustrating a false-negative AI-assisted outcome where the reader's initial assessment was correct but was overridden by a low AI confidence score. Case 3: Axial T2-WI (**i**), high b-value DWI (**j**), and ADC map (**k**) show a finding in the right anterior transition zone (dashed arrows), scored PI-RADS 3 by a basic reader on bpMRI. The AI system did not detect a focal lesion (Pi score 2.0; l), and the reader downgraded the score to PI-RADS 2. Biopsy was benign, illustrating a true-negative AI-assisted downgrade where the AI appropriately reduced an unnecessary biopsy referral. GG = grade group; WI: weighted image

In this study, AI was used as a concurrent decision support system. This usage scenario may have contributed to the drop in sensitivity seen with AI, particularly for less-experienced readers (Fig. 4). Where cancer detection is a priority, use of AI as an additional reader, or other approaches to minimise false negatives such as AI-specific training, should be considered to promote increased sensitivity.

This study has limitations. It was retrospective, single-centre, with a moderate sample size and few csPCa cases in the AI subset, so subgroup and benefit-to-harm estimates are imprecise. Histology was mainly based on targeted/systematic biopsies, with some MRI-negative men classified by follow-up alone, which likely missed a proportion of indolent or MRI-occult cancers and makes absolute sensitivity and specificity less robust than within-patient comparisons. We evaluated a single CE-marked AI tool in one vendor/protocol setting, and despite a 45-day wash-out, the fixed reading order and case reuse do not fully exclude recall. Importantly, in session 1 bpMRI was always scored before DCE unblinding within the same sitting, which increases within-reader, within-case correlation and may artificially narrow the confidence interval for the bpMRI–mpMRI AUC difference; a fully crossed design with separate sessions would have provided a more conservative estimate. Furthermore, no a priori sample size calculation was performed for the non-inferiority comparison, so the study may have been underpowered to detect a clinically meaningful difference, and the non-inferiority conclusion should be regarded as exploratory. Finally, the 10% non-inferiority margin for bpMRI versus mpMRI is clinically pragmatic but relatively wide; in light of recent level 1B multicentre evidence for bpMRI non-inferiority, the relatively high prevalence of csPCa in our referral cohort (39.9%) may limit generalisability to screening or lower-risk populations and should be considered when interpreting predictive values. Our data should be regarded as supporting that bpMRI can safely replace mpMRI in our institution rather than as definitive evidence to change practice universally [9].

In conclusion, these findings support a practical diagnostic model in which bpMRI is adopted as the standard protocol, streamlining workflows and avoiding contrast in most men without measurable loss in csPCa detection. AI can then be applied to bpMRI to increase biopsy efficiency and reduce unnecessary procedures, with the greatest benefit in less-experienced readers. Implementation should focus on maintaining sensitivity within an acceptable range by appropriate choice of AI operating thresholds, structured training on integration of AI outputs with PI-RADS, and routine monitoring of benefit-to-harm ratios rather than reliance on accuracy metrics alone. Within such a framework, AI-assisted bpMRI appears a feasible strategy to optimise the balance between csPCa detection and biopsy avoidance, particularly in settings lacking uniform subspecialist expertise.
